# The Huxley Lecture

**Published:** 1900-10-06

**Authors:** 


### The Huxley Lecture.

In connection with the Charing Cross Hospital Medical School Lord Lister, President of the Royal Society, delivered the third biennial Huxley lecture at St. Martin's Town Hall on Tuesday afternoon, the subject being "Recent Advances in Science and their Bearing on Medicine and Surgery."

Lord Lister, having first explained that he had been requested by the Council of the Medical School to make his own works the subject of the lecture, gave an account of some of his early investigations into the nature of inflammation and suppuration, which prepared him to welcome Pasteur's proof that putrefaction, like other fermentations, is caused by the growth of microbes in putrescible substances. This opened up a new problem: not the hopeless effort to exclude the oxygen of the air, which had previously been supposed to be the cause of the decomposition of organic substances, but the endeavour to to exclude mischievous microbes by means that did as little damage as possible to the tissues. More recent investigations had shown that hurtful microbes are not always putrefactive. But the principle which first guided him retained, as he believed, its full value, and the endeavour to carry it out to the best advantage had been his chief life-work.

Dr. T. H. Green proposed a vote of thanks to the lecturer, which was seconded by the Rev. Prebendary Kitto, vicar of St. Martin's, and heartily accorded.

Lord Lister, in acknowledging the vote, said that, having retired from the profession he had loved so well, it had been a great pleasure to him to be again amongst his *confrères* and say a few words to them in mutual confidence and esteem. He knew that when he addressed medical men any information which could be gained regarding the functions of the human body would be as cordially welcomed by them, even though such information were obtained at the expense of the lives of a few frogs, or even of an occasional rabbit.

